# The complete chloroplast genome of *Mazus pumilus* (Mazaceae)

**DOI:** 10.1080/23802359.2018.1524727

**Published:** 2018-10-29

**Authors:** Xiaofeng Chi, Faqi Zhang, Qingbo Gao, Rui Xing, Shilong Chen

**Affiliations:** a Key Laboratory of Adaptation and Evolution of Plateau Biota, Northwest Institute of Plateau Biology, Chinese Academy of Sciences, Xining, China;; b Qinghai Provincial Key Laboratory of Crop Molecular Breeding, Northwest Institute of Plateau Biology, Chinese Academy of Sciences, Xining, China

**Keywords:** *Mazus pumilus*, chloroplast genome, phylogenetic analysis

## Abstract

*Mazus pumilus* (N. L. Burman) Steenis is the representative species of *Mazus* mainly distributed in China. Here, we report the complete chloroplast genome sequence of *M. pumilus*. The genome was 153,149 bp in length with 106 genes comprising 79 protein-coding genes, 23 tRNA genes, and 4 rRNA genes. The overall GC content of *M. pumilus* chloroplast genome was is 37.8%. ML phylogenomic analysis suggested that *M. pumilus* forms a monophyletic group with *Lancea* which shows a close relationship with the clade of Phrymaceae, Paulowniaceae, and Orobanchaceae.

*Mazus* Lour. (ca. 35 species) is mainly distributed in East Asia, Australia, and New Zealand, and about 25 species are found in China (Wu and Raven 1998). *Mazus* was firstly placed in Scrophulariaceae (Wettstein 1891). However, the systematic position of *Mazus* was altered by recent molecular-phylogenetic studies. Beardsley and Olmstead (2002) found that *Mazus* and *Lancea* form a well-supported clade recognized as the subfamily Mazoideae belonging to the Phrymaceae. However, phylogenetic studies of Oxelman et al. (2005), Albach et al. (2009), Xia et al. (2009) and Schäferhoff et al. (2010) confirmed that *Mazus* should be placed apart from the Phrymaceae. Based on the previous molecular-phylogenetic studies, Reveal (2011) described a new family named Mazaceae which including *Mazus*, *Lancea* and *Dodartia*. Up to now, previous literature has not well revealed the phylogenetic relationship of *Mazus* and its related genus by different sequence fragments and need to be further elucidated.

In the present study, we report the completed chloroplast genomes of *Mazus pumilus* (N.L. Burman) Steenis which is the representative species of *Mazus*. *M. pumilus* was collected in Luoyang (112°26′45.2″E, 34°38′3.9″N, China) and the specimen was deposited in the Qinghai-Tibetan Plateau Museum of Biology (HNWP). The DNA was isolated from fresh leaves via the modified CTAB method (Doyle 1987). The complete chloroplast genome was sequenced at Novogene Biotech Co. (Tianjin, China) using the Illumina MiSeq platform. Genomic sequence was assembled with SOAPdenovo (Luo et al. 2012) and annotation was performed with CpGAVAS (Liu et al. 2012) by comparing with the previously reported chloroplast sequences of *Lancea* (Chi et al. 2018). The completed chloroplast genome sequences of *M. pumilus* together with 26 species from Lamiales and *Lactuca sativa* (outgroup) were aligned with MAFFT (Katoh and Standley 2013). Gblocks (Castresana 2000) was introduced to remove ambiguously aligned sites. A maximum likelihood (ML) analysis was implemented using RAxML-HPC2 on XSEDE based on the GTR + G + I nucleotide substitution model as recommended by jModelTest2 with 1000 replications.

The *M. pumilus* chloroplast genome (GenBank Accession No. MF593117) was 153,149 bp in length with a pair of inverted repeats (IR) regions (25,831 bp), a large single copy (LSC) region (84,034 bp), and a small single copy (SSC) region (17,453 bp). The GC content of the genome was 37.8%, and the GC contents of IR regions (43.1%) was higher than the LSC regions (35.7%) and SSC regions (32.1%). There were 106 predicted genes including 79 protein-coding genes, 23 tRNA genes, and 4 rRNA genes. Among the protein-coding genes, 63 were found in the LSC region, 11 were located in the SSC region, while *ndhB*, *rpl2*, *rpl23*, *rps7,* and *ycf2* were duplicated in the IR regions.

ML analysis showed that *M. pumilus* and *Lancea* species constituted one monophyletic group as Mazaceae (Figure 1). Additionally, Mazaceae showed a close relationship with Phrymaceae, Paulowniaceae, and Orobanchaceae, rather than the Scrophulariaceae. This newly reported chloroplast data not only provided genomic information for Mazaceae but also revealed the phylogenetic relationships. These data will empower genetic engineering, conservation genetics and evolutionary studies involving this taxon.

**Figure 1.**
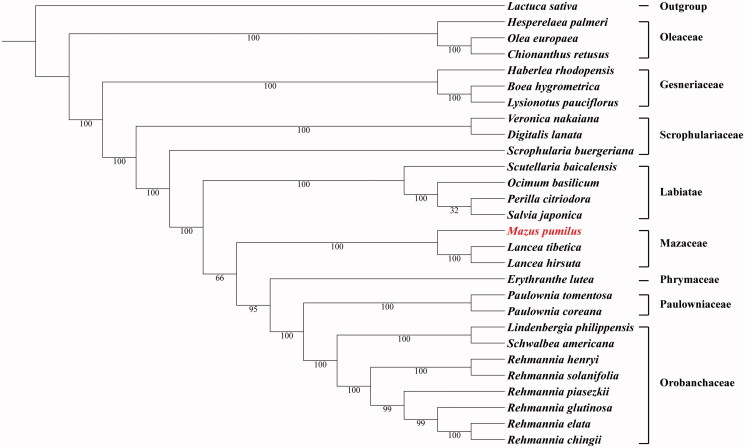
Maximum likelihood phylogenetic tree based on 28 complete chloroplast genome sequences. The number on each node indicates the bootstrap value. Accession numbers: *Boea hygrometrica* JN107811, *Chionanthus retusus* KY582962, *Digitalis lanata* KY085895, *Erythranthe lutea* KU705476, *Haberlea rhodopensis* KX657870, *Hesperelaea palmeri* LN515489, *Lactuca sativa* AP007232, *Lancea hirsuta* MG551489, *Lancea tibetica* MF593117, *Lindenbergia philippensis* HG530133, *Lysionotus pauciflorus* KX752081, *Ocimum basilicum* KY623639, *Olea europaea* GU228899, *Paulownia coreana* KP718622, *Paulownia tomentosa* KP718624, *Perilla citriodora* KT220684, *Rehmannia chingii* KX426347, *Rehmannia elata* KX636161, *Rehmannia glutinosa* KX636157, *Rehmannia henryi* KX636158, *Rehmannia piasezkii* KX636160, *Rehmannia solanifolia* KX636159, *Salvia japonica* KY646163, *Schwalbea americana* HG738866, *Scrophularia buergeriana* KP718626, *Scutellaria baicalensis* KR233163, *Veronica nakaiana* KT633216.
